# Retraction Note: Restoring the Taxol biosynthetic machinery of *Aspergillus terreus* by *Podocarpus gracilior* Pilger microbiome, with retrieving the ribosome biogenesis proteins of WD40 superfamily

**DOI:** 10.1038/s41598-026-35501-w

**Published:** 2026-01-13

**Authors:** Ashraf S. A. El-Sayed, Nabil Z. Mohamed, Samia Safan, Marwa A. Yassin, Lamis Shaban, Ahmed A. Shindia, Gul Shad Ali, Mahmoud Z. Sitohy

**Affiliations:** 1 Enzymology and Fungal Biotechnology Lab (EFBL), Botany and Microbiology Department, Faculty of Science, Zagazig University, Zagazig, 44519 Egypt; 2 Mid-Florida Research Education Center, IFAS, University of Florida, Gainesville, USA; 3 Biochemistry Department, Faculty of Agriculture, Zagazig University, Zagazig, 44519 Egypt; 4 Eukaryo Tech, LLC, Apopka, FL 32703 USA

Retraction of: *Scientific Reports* 10.1038/s41598-019-47816-y, published online 08 August 2019

The Editors have retracted this Article.

After publication, concerns were raised regarding the veracity of the following data presented in this work. Specifically,

- several patterns in the TLC profile shown in Figure 2E appear to overlap with one another;
- there are signs of undeclared splicing in Figures 5A and S2.

The Authors provided raw data on request from the Editors. However, the Editors found that the statistical data reported in Figures 1D and 2A differs from the source data provided for these experiments. The Editors therefore no longer have confidence in the research presented in this work.

Ashraf S. A. El-Sayed disagrees with the retraction. Nabil Z. Mohamed, Samia Safan, Marwa A. Yassin, Lamis Shaban, and Mahmoud Z. Sitohy did not respond to the correspondence from the Editors about this retraction. The Editors were not able to confirm the current contact details for Ahmed A. Shindia and Gul Shad Ali.

